# The Extent of the Use of GRADE in Campbell Systematic Reviews: A Systematic Survey

**DOI:** 10.1002/cl2.70082

**Published:** 2025-12-07

**Authors:** Zhenjie Lian, Rui Wang, Xuping Song, Yunze Han, Qiyin Luo, Jing Tang, Xinye Guo, Yan Ma, Yue Hu, Xufei Luo, Yaolong Chen, Kehu Yang, Howard White, Vivian Welch

**Affiliations:** ^1^ School of Public Health Lanzhou University Lanzhou China; ^2^ School of Public Health, The Centre of Evidence‐based Social Science Lanhzou University Lanzhou China; ^3^ Key Laboratory of Evidence Based Medicine & Knowledge Translation of Gansu Province Lanzhou China; ^4^ WHO Collaborating Centre for Guideline Implementation and Knowledge Translation Lanzhou China; ^5^ Department of Health Research Methods, Evidence, and Impact McMaster University Hamilton Ontario Canada; ^6^ Laiyang Center for Disease Control and Prevention Laiyang Shandong Province China; ^7^ Lanzhou Institute of Biological Products Company Limited Lanzhou China; ^8^ School of Basic Medical Science, Centre for Evidence‐Based Medicine Lanzhou University Lanzhou China; ^9^ Bruyère Research Institute Ottawa Ontario Canada

**Keywords:** Campbell systematic reviews, certainty in evidence, GRADE, summary of findings

## Abstract

**Objective:**

To conduct a systematic survey on the extent of the use of the grading of recommendations, assessment, development, and evaluations (GRADE) and other evidence grading systems in Campbell systematic reviews (SRs).

**Study Design and Settings:**

Campbell SRs published before January 1st, 2024, that used evidence grading systems were included. General characteristics and details of a summary of findings (SoF) table and an evidence profile (EP) were independently extracted by two investigators.

**Results:**

Among 234 SRs retrieved, 46 (19.66%) used evidence grading systems, all of which were GRADE. One used GRADE erroneously to rate the quality of individual studies rather than the body of evidence. The 45 SRs used GRADE to assess the certainty of a body of evidence and included 858 outcomes. Of them, the certainty in evidence for 32 were rated as high (3.73%), 170 were moderate (19.81%), 291 were low (33.92%), and 365 were very low (42.54%). Among the 1860 instances of downgrading and upgrading, the certainty in evidence was mostly downgraded for risk of bias (ROB) (1026, 55.16%) and imprecision (408, 21.94%). The large magnitude of effect (14, 0.75%) and plausible confounding (10, 0.54%) were the main upgraded factors. The proportions for higher certainty in evidence (including high and moderate) were larger in the international development (9.59%) and social welfare (7.55%) groups than in the other groups (1.37%).

**Conclusion:**

Most Campbell SRs do not assess the GRADE certainty in evidence. Where evidence is evaluated, the quality of that evidence is mainly low or very low, and this is most commonly due to serious ROB or imprecision.

## 1 Plain Language Summary

For systematic reviews (SRs), the certainty in evidence is crucial. It is essential to understand the application status of evidence grading systems in Campbell SRs.

### 1.1 The Review in Brief

Campbell's policies and guidelines propose preparing a summary of findings (SoF) table or evidence profile (EP), referring to the GRADE approach, to present the certainty in evidence.

Understanding the status of GRADE in Campbell SRs may help facilitate its usage.

### 1.2 What Is This Review About?

Compared with other types of SRs, Campbell SRs are of higher quality, are more comprehensive, and unbiased. As for SRs, the certainty in evidence reflects how confident one can be that the effect estimate is close to the true effect.

### 1.3 What Is the Aim of This Review?

This study aims to assess the extent of the use of the GRADE system in Campbell SRs.

### 1.4 What Studies Are Included?

This review identified 234 reviews published before January 1st, 2024, from Campbell Systematic Reviews on the Wiley Online Library website, of which 45 were included in the analysis.

### 1.5 What Are the Main Findings of This Review?

Forty‐five Campbell SRs, encompassing 858 outcomes, appropriately used GRADE to assess the certainty in evidence. In general, the certainty in evidence is low or very low, primarily due to the serious risk of bias (ROB) and imprecision. Reviews produced by International Development and Social Welfare groups had a higher proportion of high or moderate quality.

### 1.6 What Do the Findings of This Review Mean?

These findings can help researchers to adapt GRADE to Campbell topic areas with good feasibility and credibility.

#### 1.6.1 How Up‐to‐Date Is This Review?

This review included all the Campbell SRs published up to January 2024.

## 2 Background

### 2.1 Description of the Problem or Issue

SRs use scientific and transparent methods to search, evaluate, and synthesize the best available evidence related to a specific research question (Noonan and Bjørndal 2010; Li et al. 2015). The Campbell Collaboration aims to produce high‐quality, open, and policy‐relevant evidence syntheses and policy briefs in social science (Wei et al. 2019; Zuo et al. 2018). Compared with other types of SRs, Campbell SRs are of higher quality, are more comprehensive, and unbiased. As such, Campbell SRs can support decision‐makers seeking to develop better policies and can also improve decision‐making among individuals (Hannes et al. 2007; Wang et al. 2010). The Campbell Collaboration has 11 coordinating groups, which are responsible for the production, scientific merit, and usefulness of Campbell publications. These coordination groups are designed based on core topics in social science.

Assessing the quality of the available evidence is a prerequisite for providing the most comprehensive, reliable, and best evidence (Purssell and McCrae 2020). In 2004, a clear, comprehensive, and transparent system named GRADE (the grading of recommendations, assessment, development, and evaluations) was developed. The objective of the GRADE system is to make it easier for users to rate the certainty in evidence in SRs (Petrisor et al. 2006; Guyatt, Oxman, Akl, et al. 2011). As for SRs, the certainty in evidence reflects how confident one can be that the effect estimate is close to the true effect (Balshem et al. 2011; Guyatt et al. 2008). In addition, the “certainty of the evidence” is an assessment of the likelihood that the effect will be substantially different from what the research found (GRADE Working Group 2004). Therefore, in the GRADE system, the phrases “quality of evidence,” “certainty in evidence,” “strength of evidence,” and “confidence in estimates” are used interchangeably (GRADE Working Group 2004, 2013). This study will use the “certainty in evidence.”

The GRADE process includes defining the question, collecting evidence, rating evidence quality, and grading recommendations (Guyatt, Oxman, Akl, et al. 2011). Evidence tables, including a SoF table and an EP, are key tools for presenting the quality of the available evidence and the judgments that have a bearing on the quality rating (Guyatt, Oxman, Akl, et al. 2011; GRADE Working Group 2013). The difference between the two is that EP is intended for review authors, those preparing SoF tables, and those questioning a quality assessment. The SoF table, on the other hand, can be used by a wider audience, including the end users of SRs and guidelines (Campbell Collaboration 2020).

The GRADE system rates the certainty in evidence for each outcome, and identifies five reasons that can reduce the quality of a body of evidence, namely ROB, inconsistency, indirectness, imprecision, and publication bias and three factors that may increase quality, particularly in the context of non‐randomized studies (NRSs), namely large magnitude of effect, dose–response gradient, and effect of plausible residual confounding (Balshem et al. 2011; Guyatt, Oxman, Sultan, et al. 2011). In 2021, Campbell's policies and guidelines proposed the optional preparation of SoF or EP, referring to the GRADE approach (Campbell Collaboration 2020). In addition, Campbell has a policy that the “quality of evidence” be described, and the checklist indicates “interpretation should also consider certainty which includes the concepts of precision, inconsistency, risk of bias, including publication selection bias, and directness of the evidence.”

The GRADE system offers potential benefits to clinicians, patients, and policymakers. Physicians and patients use the GRADE system in their clinical practices, and policymakers use GRADE in the development of health policies (GRADE Working Group 2013). Meanwhile, it has been applied successfully to diagnosis, prognosis, reviews of qualitative research (CER‐QUAL), and work is being done for modeling, and it could be applied to a wide range of non‐health intervention settings. Therefore, it is necessary to learn about the application status of evidence grading systems in Campbell SRs.

### 2.2 Why It Is Important to Conduct This Review?

To date, no studies have been published that deal with the application status of evidence grading systems in Campbell SRs. The extent to which Campbell SRs used the GRADE approach remains unclear. Assessing the application status of GRADE in Campbell SRs will help researchers to adapt GRADE to Campbell topic areas with good feasibility and credibility.

## 3 Objectives

This study has two main objectives:

- To examine the extent of the use of the GRADE system in Campbell SRs.
- To describe the quality of a body of evidence for Campbell SRs, and to summarize upgrading and downgrading factors.

## 4 Methodology

### 4.1 Criteria for Considering Studies for This Review

#### 4.1.1 Types of Studies

Campbell SRs of all types and topics were included. For Campbell SRs that had been updated one or more times, the most recent version was included. Protocols, methods papers, commentaries, editorials, and other types of evidence synthesis, such as evidence maps and mega maps, were excluded.

#### 4.1.2 Types of Participants

Not applicable.

#### 4.1.3 Types of Interventions

Not applicable.

#### 4.1.4 Types of Outcome Measures

Campbell SRs that used the GRADE system to assess the certainty in evidence were included. We excluded SRs that mentioned GRADE in their methodology but did not provide either an evidence profile (SOF or EP table) or a summary of the quality of evidence in the full text.

### 4.2 Search Methods for Identification of Studies

Campbell SRs published before January 1st, 2024, were identified through a systematic search on the Wiley Online Library website. In addition, a manual search was conducted of the tables of contents of all Campbell SRs.

### 4.3 Data Collection and Analysis

#### 4.3.1 Study Screening

Two researchers independently identified all Campbell SRs that used evidence grading systems. An initial screening of titles and abstracts was performed, followed by a full‐text review. Any disagreements were resolved by a third reviewer.

#### 4.3.2 Data Extraction

Using a pre‐developed table in Microsoft Excel 2019, two researchers independently performed data extraction. The general characteristics and the details of the GRADE assessment were extracted, including the titles, authors, coordinating groups, certainty in evidence, and reasons for upgrading and downgrading. See Appendix A for details. Moreover, the term “inconsistency” here refers to an unexplained heterogeneity of results. To explore the details of the Campbell authors' specific reasons (footnotes of the evidence tables) for downgrading due to inconsistency, high‐frequency reasons were maintained for “I‐squared statistic (*I* ^2^)” and “inconsistencies in the effect size.” Due to insufficient data on the outcome, some specific outcomes for three of the included reviews were not graded (Miller et al. 2011; Liu et al. 2014; Welch et al. 2016). Elements of evidence tables were also extracted by referring to the standard Cochrane SoF table, which includes the table title and headline results, absolute effect, 95% confidence interval, and relative effect, among others.

#### 4.3.3 Data Analysis

Descriptive statistics were used to summarize the results as frequencies and percentages. This was used to report and describe the basic information of the included reviews, the use of GRADE, the distribution of factors for upgraded and downgraded domains, and the quality of the body of evidence. When one outcome was downgraded due to various factors, the potential linkages between those factors were not explored, as the footnotes did not clearly report the linkages. For example, the certainty in evidence was downgraded due to the serious ROB, but the ROB may also lead to serious inconsistencies (Wang et al. 2022). To compare the differences between groups, the evidence quality of outcomes was divided into two groups: higher (including high and moderate) certainty in evidence and lower (including low and very low) certainty in evidence (Wang et al. 2022). In addition, to analyze whether differences existed in the evidence quality of outcomes, including various study types, the outcomes were divided into three groups: (1) outcomes that only included randomized controlled trials (RCTs), (2) outcomes that only included NRSs, and (3) outcomes that included both RCTs and NRSs (RCTs/NRSs) (Lewis and Burke 1949).

## 5 Results

### 5.1 Characteristics of the Included SRs

Among the 234 SRs retrieved, 46 (19.66%) used evidence grading systems, all of which were GRADE. One SR employed an adapted version of the evidence grading system that was developed by Emile Tompa in 2007. However, we excluded this SR due to the lack of a rigorous methodological description and an identified name (Dyreborg et al. 2022; Tompa et al. 2007). The range of publication years for all SRs was from 2006 to 2023. We excluded one SR which misused GRADE to rate the quality of individual studies (Carthy et al. 2020). Therefore, we only analyzed the quality of the bodies of evidence for 45 Campbell SRs. These reviews were produced by the following six Campbell coordinating groups: social welfare (16, 35.56%), international development (9, 20.00%), education (5, 11.11%), crime and justice (3, 6.67%), ageing (1, 2.22%), and knowledge translation and implementation (1, 2.22%). In addition, 10 (22.22%) simultaneously covered two groups, as detailed in Appendix B and Appendix C.

Overall, there were 858 individual outcome ratings (Table 1). The certainty in evidence for all examined outcomes was rated according to GRADE: very low (365, 42.54%); low (291, 33.92%); moderate (170, 19.81%), and high (32, 3.73%). This study identified 1860 instances of downgrading and upgrading. ROB (1026, 55.16%), imprecision (408, 21.94%), inconsistency (324, 17.42%), indirectness (27, 1.45%), and publication bias (51, 2.74%) were the major factors influencing downgrading. Meanwhile, a large magnitude of effect (14, 0.75%) and plausible confounding (10, 0.54%) were the main factors that led to an upgrade.

**Figure 1:**
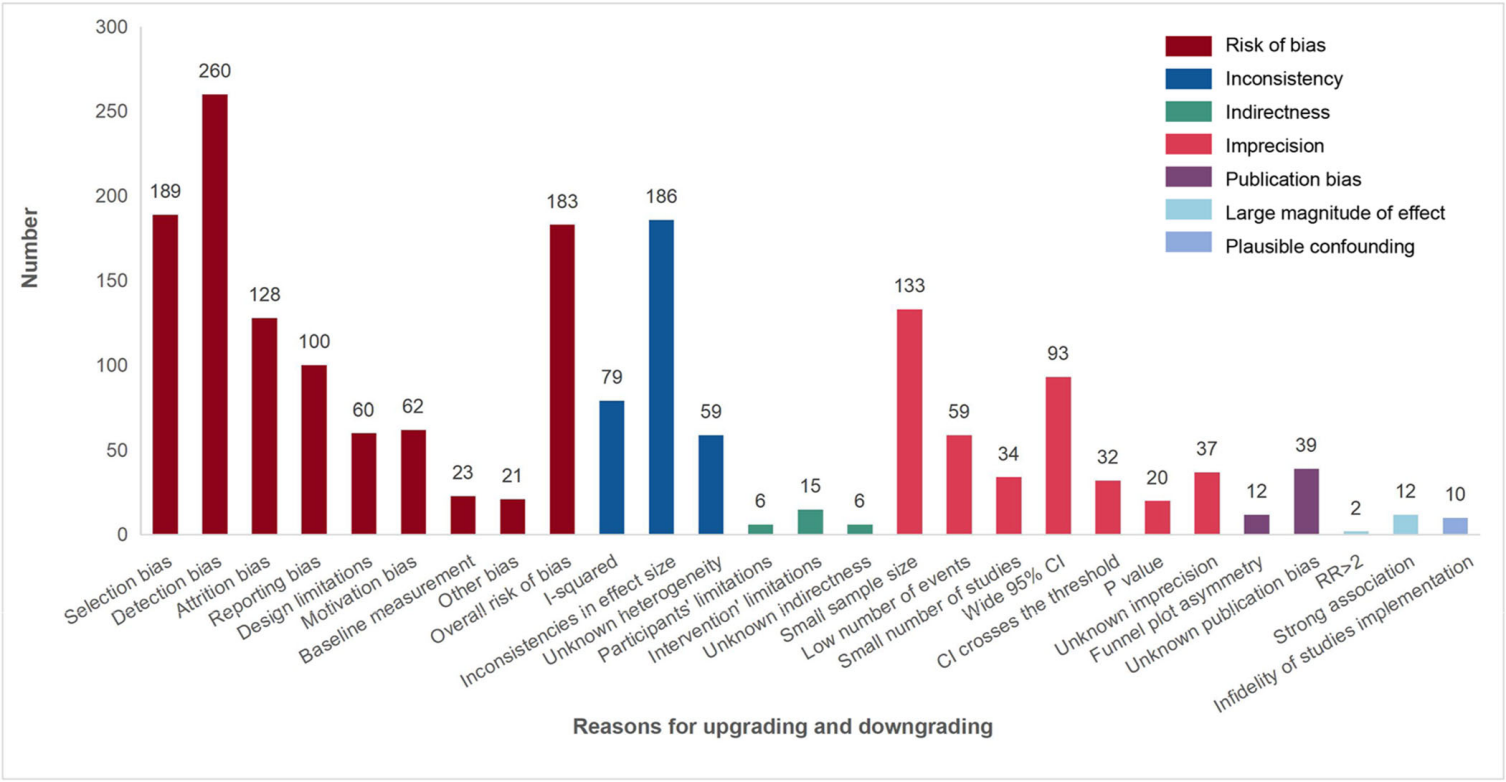
Reasons for downgrading and upgrading bodies of evidence. Overall/one or more high risk of bias domains: (1) the certainty in evidence was downgraded if one or more domains were assessed to be high or unclear risk of bias. (2) For the overall risk of bias, the authors of the reviews took into account the risk of bias from individual studies, rather than from single domains (Wang et al. 2022).

The main reason for downgrading due to ROB was detection bias (260, 25.34%), while the most common reason for downgrading due to inconsistency was inconsistencies in effect size (186, 57.41%). Of the 408 outcomes that were downgraded because of imprecision, 133 (32.60%) outcomes were because of the small sample size. Of the 45 SRs, only three described the reasons for upgrading, while 14 outcomes were upgraded because of the large magnitude of effect. The main reasons for upgrading due to the large magnitude of effect were strong linkage (12, 85.71%) and risk ratio > 2 (2, 14.29%) (Toon and Gurusamy 2014; Lwamba et al. 2022), as detailed in Figure 1.

### 5.2 The Certainty in Evidence for Different Coordination Groups

**Figure 2:**
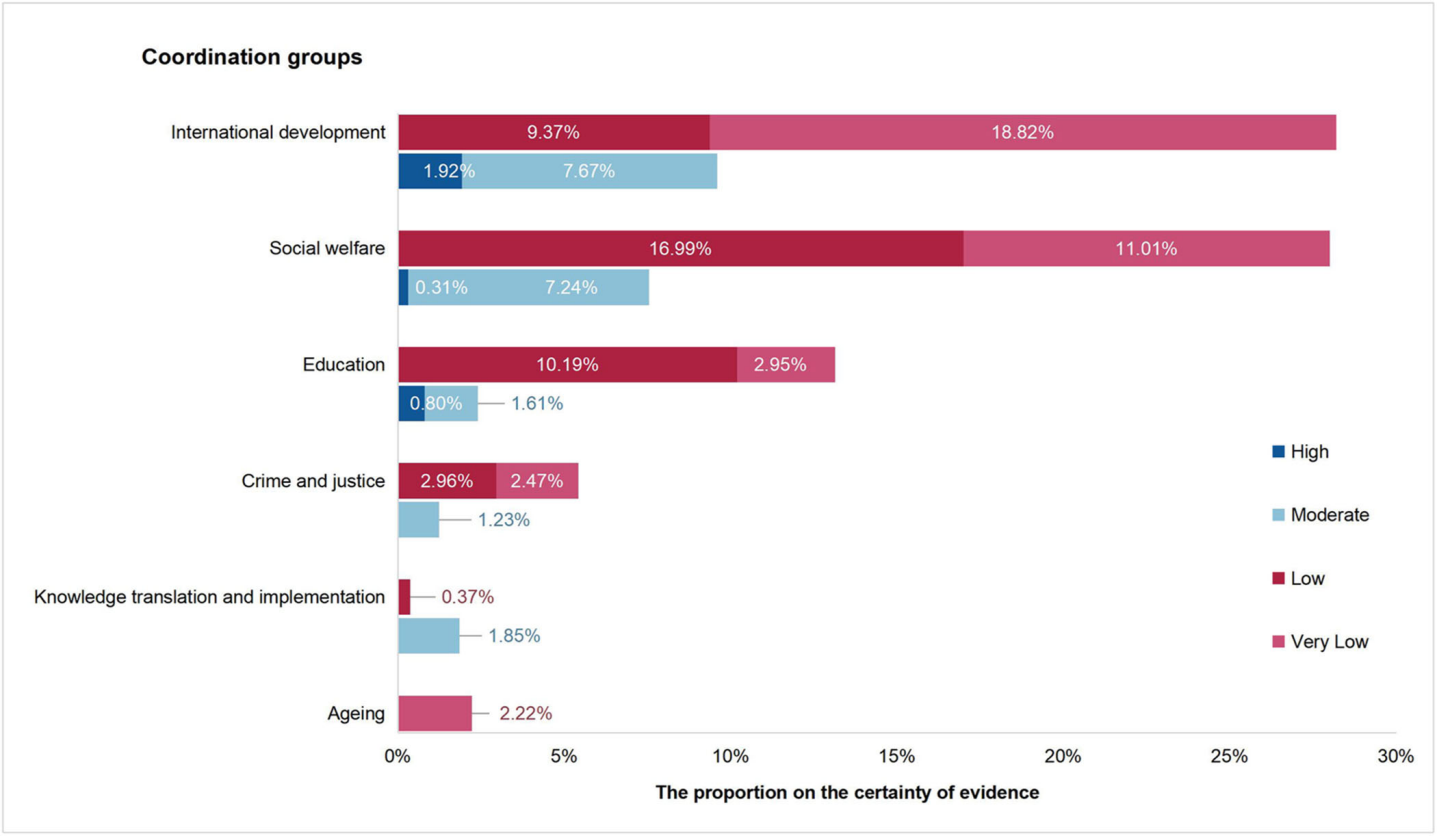
The proportion of the evidence quality in different coordination groups.

Figure 2 shows the proportion of the evidence quality in different coordination groups. For the reviews covering both groups, the analysis was performed according to the first group mentioned in Appendix B. The proportions for higher certainty in evidence (including high and moderate) were larger in the international development (9.59%) and social welfare (7.55%) groups than in the other groups (average 1.37%). With regard to downgraded domains, the weight of downgraded factors varied among Campbell coordination groups. Specifically, the ROB was the main downgraded factor in all five Campbell coordination groups, except for “ageing.” In addition, in the SRs of the crime and justice, education, and international development groups, “large magnitude of effect” and “plausible confounding” were the main factors that led to an upgrade.

### 5.3 Comparison of Details Among Different Study Design Groups

Among the 858 individual outcome ratings, 579 were based solely on RCTs (4.84% rated as high quality), 134 on NRSs (0.75% high quality), and 145 incorporated both study designs (2.07% high quality). Analysis of 1836 instances of downgrading revealed that 1289 instances were from RCT‐only results; 244 instances from NRS‐only; and 303 from both study designs. ROB constituted the primary downgrading factor across all groups. Regarding 24 instances of upgrading, 10 instances occurred in results based solely on RCTs; the results only included NRSs involved 9 instances; and the results included both study designs involved 5 instances.

### 5.4 Evidence Tables

(1) Of the 45 SRs that used GRADE to assess the certainty of a body of evidence, five used EP, and 40 used the SoF table. (2) The GRADEpro GDT is available to assist reviewing authors in the preparation of the evidence table. In this study, 12 (26.67%) SRs used the GRADEpro software to generate the evidence table; the others did not describe details on the means of producing the evidence table, so they may or may not have used GRADEpro GDT.

The detailed contents of the evidence table were varied in the 45 SRs. Specifically: (1) 25 SRs (55.56%) provided a brief description of the table title and header, including population, setting, comparison, and interventions. (2) Regarding the outcomes, the criteria provided stated that the evidence table should include up to a maximum of seven outcomes, and 34 (75.56%) eligible articles met this criterion. Meanwhile, the number of outcomes exceeded seven in the remaining 11 articles, ranging from 8 to 107. In addition, 15 (33.33%) SRs provided time frames for the measurement of the outcomes. (3) The evidence tables of 32 SRs described absolute risk, 26 SRs described the relative effect, and 19 SRs included both. (4) Two SRs did not include either the number of participants or the number of studies. The certainty in evidence was sufficiently described in the evidence tables. The comment was not described in 30 SRs, and two SRs did not describe the explanation (see Appendix A for details).

## 6 Discussion

### 6.1 SoF

The analysis shows that only 45 Campbell SRs use the GRADE system to rate the certainty in evidence of a specific outcome. The certainty in evidence for the majority of outcomes was assessed as very low or low, mainly because of the serious ROB (1026, 55.16%). Cochrane RoB is a tool that is commonly used to assess the ROB for RCTs. For ROB 1.0, a formal evaluation found some bias domains to be occasionally confusing. In particular, assessment of bias due to incomplete outcome data and selective reporting of outcomes caused particular difficulties. There was also confusion over whether studies that were not blinded should automatically be considered to be at high ROB (Sterne et al. 2019; Savović et al. 2014). Compared with ROB 1.0, ROB 2.0 more adequately addresses these problems (Savović et al. 2014). However, this study found that, in Campbell SRs, ROB 1.0 was still used more frequently than ROB 2.0. The reasons for the differences may include: (1) The ROB 2.0 assessment process is often more cumbersome and time‐consuming. (2) Researchers may also potentially misunderstand or misinterpret some new concepts in RoB 2.0. (3) There is no widely used interactive web tool for completing a RoB 2.0 assessment (Savović et al. 2014; Yang et al. 2017). Differences in the certainty of evidence and downgraded and upgraded factors were determined for different Campbell coordination groups and different study designs. Campbell researchers producing SRs also choose different evidence tables depending on their needs, and the SoF table is used much more than the EP table.

Despite existing challenges, the GRADE approach holds considerable promise for broader application in Campbell SRs. First, GRADE cannot be applied uniformly across all settings, especially when study designs vary. ROB constituted the primary downgrading factor across all groups. In Campbell reviews, the inclusion of RCTs, quasi‐experimental, and observational studies adds complexity to the assessment of ROB. Different appraisal tools are used, and the application of standard GRADE criteria becomes limited in such contexts. Second, inconsistency is particularly challenging when inconsistent effects are anticipated. Inconsistency refers to an unexplained heterogeneity of results. Patients vary widely in their pre‐intervention or baseline risk of the adverse outcomes that health care interventions are designed to prevent. This is especially relevant across the diverse populations commonly included in Campbell SRs. Third, GRADE was originally developed for health care interventions, particularly to support reviews and guidelines that examine alternative management strategies or interventions. Applying GRADE directly in the social science and social sector, where heterogeneity is often greater, presents additional challenges. Nevertheless, the GRADE approach offers a valuable reference framework for assessing the certainty in evidence in social science.

**Table 1:** Details on downgrading and upgrading for evidence with different study designs.

Items	Total (%)	RCTs (%)	NRSs (%)	RCTs/NRSs (%)
The quality of a body of evidence, *n*	858	579	134	145
High, *n* (%)	32 (3.73)	28 (4.84)	1 (0.75)	3 (2.07)
Moderate, *n* (%)	170 (19.81)	147 (25.39)	3 (2.24)	20 (13.79)
Low, *n* (%)	291 (33.92)	225 (38.86)	21 (15.67)	45 (31.03)
Very low, *n* (%)	365 (42.54)	179 (30.92)	109 (81.34)	77 (53.10)
Total number of downgrading domains, *n*	1836	1289	244	303
Risk of bias, *n* (%)	1026 (55.88)	734 (56.94)	136 (55.74)	156 (51.49)
Inconsistency, *n* (%)	324 (17.65)	191 (14.82)	52 (21.31)	81 (26.73)
Indirectness, *n* (%)	27 (1.47)	20 (1.55)	5 (2.05)	2 (0.66)
Imprecision, *n* (%)	408 (22.22)	319 (24.75)	46 (18.85)	43 (14.19)
Publications bias, *n* (%)	51 (2.78)	25 (1.94)	5 (2.05)	21 (6.93)
Total number of upgrading domains, *n*	24	10	9	5
Large effect, *n* (%)	14 (58.33)	4 (40.00)	8 (88.89)	2 (40.00)
Dose‐response, *n* (%)	0	0	0	0
Plausible confounding, *n* (%)	10 (41.67)	6 (60.00)	1 (11.11)	3 (60.00)
Mean frequency, *n* of	2.14	2.23	1.82	2.09
Downgraded domains, *n*/Outcomes rated, *n*	1836/858	1289/579	244/134	303/145
Mean frequency, *n* of	0.03	0.02	0.07	0.03
Upgraded domains, *n*/Outcomes rated, *n*	24/858	10/579	9/134	5/145

Abbreviations: NRSs, non‐randomized studies; RCTs, randomized controlled trials.

### 6.2 Application Status of Evidence Table

Evidence tables, including EP and SoF tables, are widely used to present the main findings of a review in a transparent, structured, and simple tabular format. Both the GRADE handbook and the Cochrane handbook for systematic reviews of interventions require that an evidence table (SoF/EP) be used as a key tool in the presentation of evidence, the corresponding results, and the results of grading the certainty in evidence (GRADE Working Group 2013; Higgins et al. 2023). The elements of the evidence table are the manifestation of the grading approach and evidence summary process (Guyatt, Oxman, Akl, et al. 2011). Chapter 14 of the Cochrane Handbook introduces readers to a general template for an evidence table. An evidence table can be correctly produced using GRADE's software, GRADEpro GDT (Mulrow and Oxman 1996). However, some problems remain with regard to the description of elements in the standard Cochrane evidence table format. For example, in the SR by Lwamba et al. the EP framework was incorrectly labeled as a SoF table (Lwamba et al. 2022). In the future, users should read the GRADE handbook along with a series of articles that describe the elements of the GRADE evidence table to improve their understanding, find key information, and understand the differences in different versions (Campbell Collaboration 2020; Carrasco‐Labra et al. 2016; Langendam et al. 2016; Santesso et al. 2016). In short, the correct format of the evidence table should be chosen according to different purposes and different audiences.

### 6.3 Strengths and Limitations

In this study's literature review, no articles were found that examine the use of evidence grading systems in the Campbell SRs. To the best of our knowledge, this study comprehensively surveys the use of evidence grading systems in Campbell SRs.

This study also has some limitations. For one thing, the reasons for repeated downgrades are not explored, as the linkage between the downgrades is not stated in the footnotes. The detailed reasons why Campbell's authors did not use an evidence grading system are also not explored; these will be pursued in the future with the Campbell methods coordinating group (Aloe et al. 2024). The usability or applicability of the GRADE approach to Campbell topic areas was not assessed. The health care intervention reviews often have fewer studies and narrower questions than Campbell reviews, such that the directness of the intervention and population may be easier to assess (Moher et al. 2007; Page et al. 2016). However, other social science fields also urgently need generally recognized evidence grading tools to assess the quality of a body of evidence. In the future, it is hoped that by assessing the usability or applicability of the GRADE approach in the Campbell SRs, certain references can be provided for the adaptation of GRADE in the field of social sciences. In addition, a protocol for this study was not published, which could be a limitation.

### 6.4 Findings From Other Studies

A study by Ge et al. used a cross‐sectional survey to investigate the extent of the use of the GRADE approach in Cochrane reviews of traditional Chinese medicine. The common downgrade factors were mainly serious ROB and imprecision (Wang et al. 2022). A study by Julian Eble et al. assessed the use of GRADE in the evidence syntheses published in high‐impact‐factor nutrition journals. Certainty in evidence was downgraded mainly because of the RoB and imprecision in the SRs of RCTs, as well as for imprecision, RoB, and inconsistency in the SRs of NRSs (Werner et al. 2021). In 2014, the GRADE working group created the environmental and occupational health project group to begin focusing on the environmental field (Morgan et al. 2019). The systematic survey results of this study revealed that the quality of the evidence is generally low to very low, and concentration‐response gradient and the large magnitude of an effect were the most common upgrade factors (Song et al. 2023).

### 6.5 Implications of GRADE

#### 6.5.1 Implications for Practice

For authors using GRADE for Campbell reviews, detailed guidelines are available to help create concise, clear, accurate, and relevant explanatory footnotes and comments (Mulrow and Oxman 1996; Carrasco‐Labra et al. 2016). Furthermore, it is necessary to provide training in GRADE and establish effective feedback channels to assist researchers in applying GRADE. For Campbell to improve the application of the GRADE system, editors could use these guidelines to facilitate the implementation of GRADE.

#### 6.5.2 Implications for Research

Campbell researchers may consider additional factors to be important in judging quality, such as funding by industry. This is because many Campbell sectors are driven by branded programmers, where the intervention studies are designed and carried out by the people in the branded program who organized and created the program (Sanders et al. 2014). Industry funding has been identified as a potential source of bias, but is not included in Cochrane ROB 1.0 or ROB 2.0, though it has been identified as needing further research. For Campbell reviews, where branded programs are evaluated by the organization that developed the program, there may be a need to research methods to explicitly consider source of funding in rating the certainty of evidence. Research is needed on applying the GRADE system to different sectors, the system's feasibility and credibility, as well as whether additional valuable domains for upgrading and downgrading.

## 7 Conclusion

Most Campbell reviews do not assess the GRADE certainty in evidence. Where evidence is evaluated, the quality of that evidence is mainly low or very low, and this is most commonly due to the serious ROB or imprecision. The rate of high or moderate quality was higher in reviews produced by the International Development and Social Welfare groups.

## Author Contributions

**Zhenjie Lian:** writing – original draft (lead), data curation (equal), formal analysis (equal), writing – review and editing (equal). **Rui Wang:** writing – original draft (lead), data curation (equal), formal analysis (equal), writing – review and editing (equal). **Xuping Song:** conceptualization (lead), methodology (lead), formal analysis (lead), writing – original draft (equal), formal analysis (equal), writing – review and editing (equal). **Yunze Han:** writing – original draft (lead), data curation (lead), formal analysis (supporting), writing – review and editing (equal). **Qiyin Luo:** writing – original draft (supporting), writing – review and editing (equal). **Jing Tang:** writing – original draft (supporting), writing – review and editing (equal). **Xinye Guo:** writing – original draft (supporting). **Yan Ma:** writing – original draft (supporting). **Yue Hu:** writing – original draft (supporting). **Xufei Luo:** methodology (supporting). **Yaolong Chen:** conceptualization (supporting); methodology (supporting), supervision (equal). **Kehu Yang:** conceptualization (supporting), methodology (supporting), supervision (equal). **Howard White:** conceptualization (supporting), methodology (supporting), supervision (equal). **Vivian Welch:** conceptualization (supporting), methodology (supporting), writing – review and editing (equal), supervision (equal).

## Conflicts of Interest

Currently, one author (Vivian Welch) is serving as editor in chief of Campbell Systematic Reviews. She was not involved in the data collection or assessment of these reviews. The other authors declare no conflicts of interest.

## Plans for Updating This Review

Considerations will be given to conducting an update of this method's assessment 5 years after the date of publication.

## Supporting information

Appendix A The details of evidence tables.

Appendix B Basic characteristics of included SRs.

Appendix C The list of CGs.

## Data Availability

All references included in the full text are publicly available. The data sets used and/or analyzed during the current study are available from the corresponding author on reasonable request.
