# Supplementary material for: The Extent of the Use of GRADE in Campbell Systematic Reviews: A Systematic Survey
Source: Campbell Syst Rev. 2025 Dec 7;21(4):e70082. doi: 10.1002/cl2.70082 (PMC12682206; doi:10.1002/cl2.70082)
Supplement: Supplementary file 2 — Appendix B Basic characteristics of included SRs. [file CL2-21-e70082-s002.docx]

Appendix B Basic characteristics of included SRs.

| **No.** | **Study** | **Campbell Coordination Groups** | **GRADE certainty of** **evidence (No. of bodies of evidence)** | | | | | **Evidence tables** |
| --- | --- | --- | --- | --- | --- | --- | --- | --- |
|  |  |  | **Total** | **High** | **Moderate** | **Low** | **Very low** |  |
| 1 | Smedslund, 2006 | Social welfare | 4 | 0 | 0 | 0 | 4 | EP |
| 2 | Coren, 2010 | Social welfare | 7 | 0 | 0 | 0 | 7 | SoF |
| 3 | Smedslund, 2011 | Crime and justice | 18 | 0 | 5 | 11 | 2 | SoF |
| 4 | Reichow, 2012 | Education, Social welfare | 7 | 0 | 0 | 7 | 0 | SoF |
| 5 | Miller, 2012 | Social welfare | 1 | 0 | 0 | 0 | 1 | SoF |
| 6 | Fellmeth, 2013 | Social welfare | 5 | 0 | 5 | 0 | 0 | SoF |
| 7 | Grant, 2014 | Social welfare | 6 | 1 | 3 | 1 | 1 | SoF |
| 8 | Toon, 2014 | Crime and justice | 8 | 0 | 0 | 0 | 8 | SoF |
| 9 | Waddington, 2014 | Education, International Development | 9 | 0 | 0 | 7 | 2 | SoF |
| 10 | Reichow, 2014 | Education | 12 | 0 | 0 | 12 | 0 | SoF |
| 11 | Filges, 2015 | Social welfare | 4 | 0 | 1 | 1 | 2 | EP |
| 12 | Barlow, 2015 | Social welfare | 12 | 0 | 0 | 6 | 6 | SoF |
| 13 | Wollscheid, 2015 | Social welfare | 24 | 0 | 0 | 21 | 3 | SoF |
| 14 | Kristjansson, 2015 | International Development, Social Welfare | 13 | 0 | 8 | 0 | 5 | SoF |
| 15 | Polec, 2015 | International development | 5 | 0 | 4 | 1 | 0 | SoF |
| 16 | Walsh, 2015 | Social welfare | 5 | 0 | 5 | 0 | 0 | SoF |
| 17 | Welch, 2016 | International development | 17 | 4 | 4 | 6 | 3 | SoF |
| 18 | Rivas, 2016 | Social welfare | 13 | 0 | 6 | 3 | 4 | SoF |
| 19 | Coren, 2016 | International development | 6 | 0 | 4 | 2 | 0 | SoF |
| 20 | Smedslund, 2017 | Social welfare | 23 | 0 | 1 | 18 | 4 | SoF |
| 21 | Buck, 2017 | International development | 34 | 0 | 8 | 18 | 8 | EP |
| 22 | Marx, 2017 | Education | 6 | 0 | 0 | 0 | 6 | SoF |
| 23 | Vibe, 2017 | Social welfare | 12 | 0 | 10 | 2 | 0 | SoF |
| 24 | Munthe-Kaas, 018 | Social welfare | 63 | 0 | 5 | 31 | 27 | SoF |
| 25 | Petkovic, 2018 | Knowledge translation and implementation | 6 | 0 | 5 | 1 | 0 | SoF |
| 26 | Harada, 2019 | Crime and justice | 1 | 0 | 0 | 1 | 0 | SoF |
| 27 | Snilsveit, 2019 | International development | 8 | 0 | 0 | 4 | 4 | EP |
| 28 | Salam, 2019 | International development | 5 | 0 | 5 | 0 | 0 | SoF |
| 29 | Welch, 2019 | International Development, Social Welfare | 9 | 0 | 9 | 0 | 0 | SoF |
| 30 | Das, 2020 | International Development, Social Welfare | 32 | 4 | 14 | 13 | 1 | SoF |
| 31 | Salam, 2020 | International Development, Social Welfare | 6 | 0 | 0 | 2 | 4 | SoF |
| 32 | Littell, 2021 | Social welfare | 9 | 1 | 4 | 4 | 0 | SoF |
| 33 | Keats, 2021 | International Development, Social Welfare | 16 | 7 | 6 | 2 | 1 | SoF |
| 34 | Moledina, 2021 | Social welfare | 32 | 0 | 6 | 21 | 5 | EP |
| 35 | Imdad, 2021 | International Development, Social Welfare | 7 | 7 | 0 | 0 | 0 | SoF |
| 36 | Lassi, 2021 | International Development, Social Welfare | 14 | 0 | 2 | 3 | 9 | SoF |
| 37 | Lassi, 2021 | International Development, Social Welfare | 9 | 0 | 2 | 5 | 2 | SoF |
| 38 | Lwamba, 2022 | International development | 201 | 5 | 35 | 57 | 104 | SoF |
| 39 | Psaki, 2022 | International development | 144 | 0 | 5 | 19 | 120 | SoF |
| 40 | Naing, 2023 | Education | 6 | 0 | 0 | 5 | 1 | SoF |
| 41 | Littell, 2023 | Social welfare | 6 | 0 | 0 | 0 | 6 | SoF |
| 42 | Randolph, 2023 | Education | 11 | 3 | 5 | 3 | 0 | SoF |
| 43 | Dewidar, 2023 | International development | 6 | 0 | 2 | 0 | 4 | SoF |
| 44 | Laermans, 2023 | Ageing | 9 | 0 | 0 | 0 | 9 | SoF |
| 45 | McCartan, 2023 | Education | 7 | 0 | 1 | 4 | 2 | SoF |
