# Supplementary material for: The Extent of the Use of GRADE in Campbell Systematic Reviews: A Systematic Survey
Source: Campbell Syst Rev. 2025 Dec 7;21(4):e70082. doi: 10.1002/cl2.70082 (PMC12682206; doi:10.1002/cl2.70082)
Supplement: Supplementary file 3 — Appendix C The list of CGs. [file CL2-21-e70082-s001.docx]

Appendix C The list of CGs.

| **Coordination group** | **Number of published reviews** | **Number of reviews using GRADE** | **% of that CG's reviews using GRADE** |
| --- | --- | --- | --- |
| Ageing | 1 | 1 | 100.00% |
| Business and Management | 1 | 0 | 0.00% |
| Crime and Justice | 62 | 3 | 4.84% |
| Disability | 6 | 0 | 0.00% |
| Education | 49 | 7 | 14.29% |
| International Development | 43 | 17 | 39.53% |
| Knowledge Translation and Implementation | 1 | 1 | 100.00% |
| Social Welfare | 71 | 16 | 22.54% |

Footnotes: CGs, Coordination Groups.
